# Whole-Genome Sequences of 13 Chinese Indigenous Pinewood Nematodes, *Bursaphelenchus xylophilus*

**DOI:** 10.3390/ijms251910492

**Published:** 2024-09-29

**Authors:** Bo Dong, Hao Wu, Debin Li, Zaiquan Luo, Shan He, Xin Hao, Junxin Gao

**Affiliations:** 1 College of Life Engineering, Shenyang Institute of Technology, Shenfu Reform and Innovation Demonstration Zone, Shenyang 113122, China; dongbo03200410@163.com (B.D.); lidebin8096@163.com (D.L.); 13709849272@163.com (Z.L.); heshan941215@163.com (S.H.); 2 Key Laboratory of National Forestry and Grassland Administration on Northeast Area Forest and Grass Dangerous Pest Management and Control, Shenyang 113122, China; 3 Liaoning Provincial Key Laboratory of Dangerous Forest Pest Management and Control, Shenyang 113122, China; 4 Key Laboratory of Forest Disaster Warning and Control in Yunnan Province, College of Forestry, Southwest Forestry University, Kunming 650224, China

**Keywords:** pinewood nematode, whole-genome sequencing, adaptation, genetic variation

## Abstract

The pinewood nematode (*Bursaphelenchus xylophilus,* PWN) induces pine wilt disease in Pinaceae plants, causing severe destruction to pine forests. Previous studies report that by 2023, 663 cities across 18 provinces in China had been infested by the PWN, necessitating immediate control measures. To identify the genetic variations associated with the PWN’s adaptation to new environments, we conducted whole-genome sequencing (WGS) on 13 indigenous PWN samples from two distinct geographic zones within China, specifically Anhui, Liaoning, and Jiangxi provinces. We identified genetic variants and analyzed the genetic structure of these populations, followed by functional gene enrichment analyses. Our findings reveal genetic variants associated with secretion, immune system function, membrane processes, metabolism, catabolism, and cell wall regulation, supporting the hypothesis that the PWN genome has been shaped by local ecosystems.

## 1. Introduction

The pinewood nematode (*Bursaphelenchus xylophilus,* PWN) can cause pine wilt disease in Pinaceae plants, resulting in devastating damage to pine forests. It is often referred to as the “cancer” of pine trees and poses a severe threat to the ecological safety of coniferous forests across the Eurasian continent. Due to its significant detrimental impact on plant ecology and social value, the PWN has been classified as a quarantine pest by multiple countries [1]. Previous studies indicate that as of 2023, 663 cities across 18 provinces in China have been infested by the PWN (National Forestry and Grassland Administration No. 4 of 2024). Research has shown that understanding the pathways by which invasive species spread is crucial for achieving control over such species [2]. Moreover, inferring invasion pathways provides information about the process of biological invasion, enabling us to understand the ecological characteristics and dispersal trajectories of invasive populations, which is beneficial for controlling or eradicating invasive species [3]. Additionally, the PWN exhibits tolerance to both high and low temperatures. When pathogens enter a new temperature zone, they can mobilize their phenotypic plasticity to adapt to the local climate and exhibit stable genetic variation [4]. In recent years, there has been a trend in PWN expansion towards the northeastern regions, posing a significant threat to the extensive pine forests in those areas. For example, several regions in Liaoning province have reported the presence of PWN.

During the process of adapting to new environments, the PWN undergoes high levels of genetic variation through founder effects and horizontal gene transfer [5]. With the publication of the PWN genome, researchers have begun to study the genetic diversity among different PWN populations to gain new insights into this devastating disease [3,6,7]. Single-nucleotide polymorphisms (SNPs), as the most common type of genetic variation, are widely used in the study of genetic diversity and population structure in nematodes and insects [8,9,10]. In the field of nematology, SNP markers help with the identification of genetic differences between populations, improve our understanding of evolutionary relationships, and help us to track the spread of invasive species [3,9,11]. Similarly, in entomology, SNPs are employed to investigate the genetic basis of traits such as resistance to pesticides, adaptation to different environmental conditions, and the evolutionary dynamics of insect populations [12,13,14]. The application of SNP technology thus provides a powerful means to unravel the genetic mechanisms underlying the adaptability and invasiveness of the PWN.

To understand the sources of spread and population differentiation of the PWN in district geographic climate regions such as Jiangxi, Anhui, and Liaoning, genetic variants employed to analyze the population genetic structure of the PWN in these geographic areas. The detected SNP differences may help elucidate the potential selective pressures of the PWN in the aforementioned regions. Our research will provide extensive genomic information on the PWN, offering a theoretical foundation for the control and monitoring of this pest in the three provinces and for revealing the spread trajectories of the PWN in China.

## 2. Results

### 2.1. SNP Distribution in the Chinese Indigenous Pinewood Nematodes

**Figure 1:**
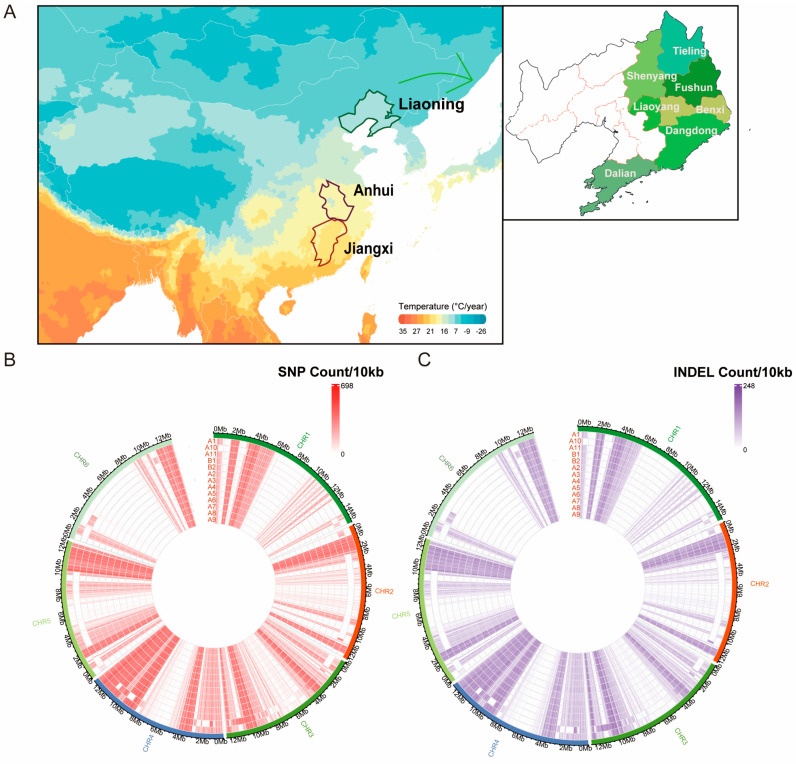
Pinewood nematode (*Bursaphelenchus xylophilus*, PWN) sampling and SNP/Indel distribution. (**A**) Geographic locations of 13 PWN samples included in this study. The map images were created by authors using https://impactlab.org/map (accessed on 1 August 2024). Detailed information about the sampling regions is provided in Table 1. (**B**) Single-nucleotide polymorphisms (SNPs) density plot. The horizontal axis shows the chromosome length (10 kb); different colors depict SNP density. (**C**) Insertions/deletions (Indels) density plot. The horizontal axis represents chromosome length (10 kb), with different colors depicting Indel density.

A total of 13 Chinese indigenous PWN samples were collected from different geographic zones for this study. These samples were sourced from three provinces (Anhui, Liaoning, and Jiangxi) and two geographic regions (temperate and subtropical) (Figure 1A). The extracted DNA underwent whole-genome re-sequencing, generating approximately 58.8 Gb of Illumina short-read data at an average coverage of 69× (Supplementary File S1).

**Table 1:** Information on samples from indigenous Chinese populations of PWN (*n* = 13).

IDs	Species	Host Tree	Insect Vector	Climate Conditions	Geographic Region (District, Province)	No. of Samples
A1	*Bursaphelenchus xylophilus*	*Pinus sylvestris* var. *mongholica*	Monochamus	Cold temperate	Hunnan, Shenyang, Liaoning	1
A2	*Bursaphelenchus xylophilus*	*Pinus tabuliformis*	Monochamus	Cold temperate	Qingyuan, Fushun, Liaoning	1
A3	*Bursaphelenchus xylophilus*	*Pinus tabuliformis*	Monochamus	Cold temperate	Dongzhou, Fushun, Liaoning	1
A4	*Bursaphelenchus xylophilus*	*Pinus tabuliformis*	Monochamus	Cold temperate	Xihu, Benxi, Liaoning	1
A5	*Bursaphelenchus xylophilus*	*Pinus tabuliformis*	Monochamus	Cold temperate	Mingshan, Benxi, Liaoning	1
A6	*Bursaphelenchus xylophilus*	*Pinus koraiensis*	Monochamus	Cold temperate	Kaiyuan, Tieling, Liaoning	1
A7	*Bursaphelenchus xylophilus*	*Pinus tabuliformis*	Monochamus	Cold temperate	Tieling, Tieling, Liaoning	1
A8	*Bursaphelenchus xylophilus*	*Pinus tabuliformis*	Monochamus	Cold temperate	Dengta, Liaoning, Liaoning	1
A9	*Bursaphelenchus xylophilus*	*Pinus tabuliformis*	Monochamus	Cold temperate	Liaoyang, Liaoning	1
A10	*Bursaphelenchus xylophilus*	*Pinus koraiensis*	Monochamus	Cold temperate	Fengcheng, Dandong, Liaoning	1
A11	*Bursaphelenchus xylophilus*	*Pinus thunbergii*	Monochamus	Cold temperate	Changhai, Dalian, Liaoning	1
B1	*Bursaphelenchus xylophilus*	*Pinus massoniana*	Monochamus	Subtropic	Yingtan, Jianxi	1
B2	*Bursaphelenchus xylophilus*	*Pinus massoniana*	Monochamus	Subtropic	Anshan, Anhui	1
Total						13

SNP calling against the BXYJv5 *Bursaphelenchus xylophilus* reference genome identified a total of 65,636 variants, comprising ~62.8 thousand SNPs and ~2.7 thousand insertions/deletions (Indels). Annotation based on genomic locations is provided in Supplementary File S2. These SNPs and Indels were evenly distributed across the genome (Figure 1B,C). SNP density is depicted as a heatmap, with darker red regions indicating areas of the genome where higher concentrations of SNPs are observed, potentially reflecting regions of increased genetic variation. Similarly, the circular plot displays the density of Indels across the same chromosomes, represented by shades of purple. These heatmaps highlight regions where Indels are more frequent, which may point to areas under specific evolutionary pressures or historical events that contributed to genetic diversity in PWN populations.

### 2.2. Genetic Relationship among Chinese Indigenous Pinewood Nematodes

**Figure 2:**
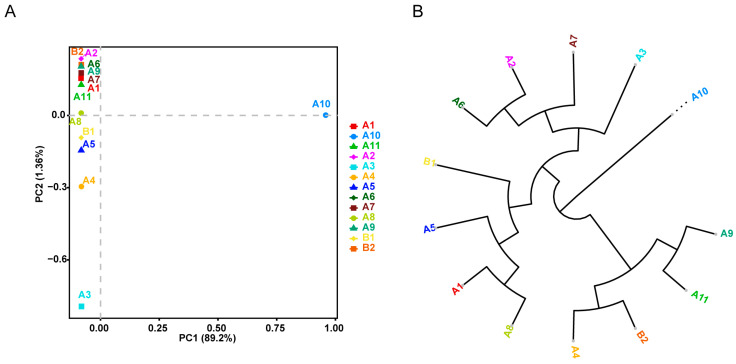
Genetic structure of Chinese indigenous PWN. (**A**) Principal component analysis (PCA) showing PC1 versus PC2 among Chinese indigenous PWN samples from different regions. (**B**) Neighbor-joining tree constructed using whole-genomic SNP data (*n* = 13).

To explore the genetic relationships among 13 Chinese indigenous PWN samples, ~62.8 K autosomal SNPs were identified. Principal component analysis (PCA) of the SNPs revealed significant differentiation, with PC1 distinguishing sample A10 from the others (Figure 2A). PC2 indicated differentiation between samples A3–5 and samples A1, A2, A6, A7, and A9 (Figure 2A). Notably, the geographic locations of samples A3–5 and A10 are all close to the ocean (Fengcheng, Fushun, and Benxi). The phylogenetic tree further demonstrated a large genetic distance between A10 and the other samples (Figure 2B). Both the PCA and the phylogenetic tree show that A10 is genetically distant from the other individuals. This distinct positioning indicates that A10 could be classified as an outgroup, suggesting that it may represent an ancestral or diverged population, distinct from the main genetic clusters formed by the other individuals.

### 2.3. Annotation of Genetic Variants and Associated Genes

**Figure 3:**
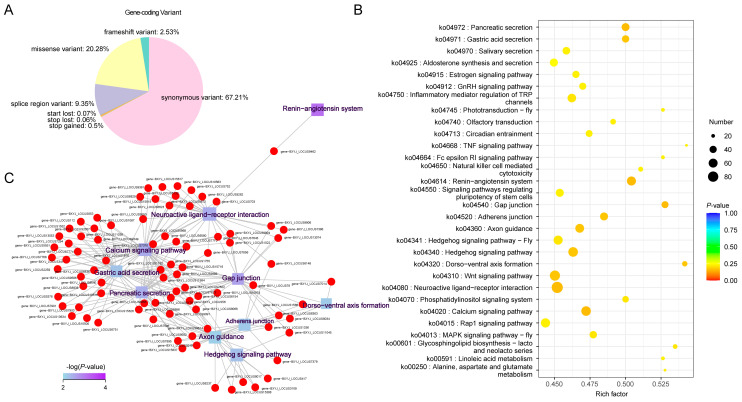
Distribution and annotation of SNP/Indel variants in Chinese indigenous PWN. (**A**) Distribution of gene-coding variant classes. (**B**) Genes and Genomes (KEGG) pathway enrichment scatter plots display significantly enriched pathways. (**C**) The KEGG pathway enrichment network illustrates the interconnections between associated genes. (*p*-value < 0.05).

**Figure 4:**
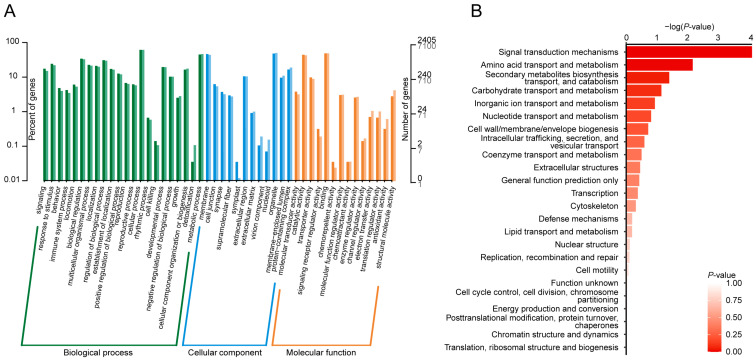
Functional annotation of genetic variants in Chinese indigenous PWN. (**A**) Gene Ontology (GO) functional analysis shows the classification of genetic variants into different biological processes. (**B**) Clusters of Orthologous Groups (KOG) enrichment analysis identifies key functional categories in which genetic variants are significantly enriched (*p*-value < 0.05).

Annotation information for gene-coding variants (SNPs and Indels), categorized by their genomic locations and variant types, is presented in Figure 3A and Supplementary File S3. The majority of gene-coding variants were located in synonymous (67.2%) and missense (20.3%) regions, while 3.2% were exonic and 9.3% were found in splice regions. To gain a comprehensive understanding of the molecular functions of the candidate SNPs/Indels identified in Chinese indigenous PWN, we performed Kyoto Encyclopedia of Genes and Genomes (KEGG) pathway, Gene Ontology (GO), and Clusters of Orthologous Groups (KOG) enrichment analyses. The KEGG analyses revealed significant enrichment in pathways associated with pancreatic secretion, gastric acid secretion, salivary secretion, calcium signaling, neuroactive ligand–receptor interactions, gap junctions, adherens junctions, and axon guidance (Figure 3B,C, Supplementary File S4). GO analysis showed that the most enriched pathways (top five) included the cellular process, metabolic process, membrane, organelle, and binding processes (Figure 4A, Supplementary File S5). KOG analysis identified significant enrichment in signal transduction mechanisms, amino acid transport and metabolism, secondary metabolite biosynthesis, transport and catabolism, carbohydrate transport and metabolism, and inorganic transport and metabolism (Figure 4B, Supplementary File S6).

### 2.4. Specific Genetic Variants in Cold-Temperate Regions

Given that the PWN was initially invasive in the warmer regions of China, we compared genetic variants between populations from cold-temperate regions (Liaoning province) and subtropical regions (Anhui and Jiangxi provinces). To minimize false positives, we included genetic variants (SNPs/Indels) that were only present in the colder region in at least three samples (MAF > 0.1), totaling 300 variants (228 SNPs and 72 Indels). Detailed information about the annotation and GO analysis is provided in Supplementary File S7. Based on their genomic location and high-impact variant type, a total of 51 variants (1 SNP and 50 Indels) were identified (Supplementary File S8). Relevant gene information for these high-impact variants is summarized in Table 2.

In the cold-temperate populations, several annotated high-impact genetic variants were associated with processes such as genetic information processing (*Bm1-29045*), metabolism (*Vha-12*, *Ugt-58*, and *SETMAR*), environmental information processing (*Vha-12*), and cellular functions (*Vha-12*). These variants may play a key role in facilitating environmental adaptation.

## 3. Discussion

In this study, we first sampled and analyzed whole-genome sequencing (WGS) data from 13 indigenous PWN samples collected from three provinces (Anhui, Liaoning, and Jiangxi) in China, representing both cold-temperate and subtropical regions. Then, we identified genetic variants and examined the population structure of these PWN samples. Additionally, we performed functional gene enrichment analyses that linked the genetic variants to key biological processes, including secretion, immune response, membrane function, metabolism, catabolism, and cell wall regulation. Last, several high-impact genetic variants and putative candidate genes were identified in cold-temperate PWNs, such as *Bm1-29045* (which is involved in genetic information processing), *Vha-12*, *Ugt-58*, and *SETMAR* (which are involved in metabolism and environmental responses). Together, these genetic results support our hypothesis that the present PWN genome might have been shaped by local ecosystems.

Since the PWN was introduced in Nanjing in 1982, this species has spread rapidly across China, exhibiting remarkable climate tolerance [15]. The PWN has been shown to thrive in environments ranging from temperate North America to subtropical Asia and Mediterranean Europe [16,17]. Previous studies predicted that PWNs’ survival was restricted to regions with maximum summer temperatures between 23 °C and 37 °C, indicating that low temperatures were insufficient for survival [18]. However, the rapid spread of pine wilt disease in northern China challenges the traditional understanding of the nematode’s potential distribution [19]. For instance, Liaoning, a province in northern China, experiences an average annual temperature of 10.74 °C (51.33 °F), with winter temperatures as low as −26 °C (−14.8 °F). This observation of PWN in Liaoning suggests that PWN has undergone genetic and physiological adaptations that enable survival in colder climates, as supported by studies indicating that PWN harbors genes and epigenetic modifications that enhance its ability to withstand environmental stressors, including low temperatures [5,20]. Therefore, we hypothesize that selection pressures may have shaped today’s local PWN genome in different environmental regions.

Genetic responses to selection pressures are often linked to changes in genetic variants [21]. Considering the PWN’s predisposition toward warm regions and its rapid adaptation to harsh environments, our study aimed to identify genetic variants within distinct ecosystems (subtropical and cold-temperate). As a result, approximately 65.6 K genetic variants were identified, with the density of variants non-uniformly distributed across the genome, likely reflecting evolutionary or environmental pressures specific to certain genomic regions. The genetic structure of 13 indigenous PWN samples was examined using 62.8 K SNPs. PCA revealed slight genetic differentiation among the samples, with the notable exception of sample A10, which exhibited significant genetic divergence along the PC1 axis. This genetic distinction was further corroborated by phylogenetic analysis, where A10 branched separately from the main cluster. The unique genetic positioning of A10 suggests it may represent an outgroup. Additionally, since A10 was collected from Fengcheng District, Dandong City, Liaoning Province, a region geographically close to the North Korean border, it is plausible that this sample represents a distinct population, which may have migrated from neighboring areas, further reinforcing its classification as an outgroup.

Integrating three different functional annotation analyses (KEGG, GO, and KOG), revealed several significant pathways potentially involved in PWN behavior, infection strategy, and environmental adaptation. KEGG analysis revealed enrichment in pathways such as pancreatic and salivary secretion, calcium signaling, neuroactive ligand–receptor interactions, and gap junctions, which are critical for PWN’s parasitism. These pathways likely facilitate cellular communication, tissue invasion, and host manipulation, with secretion-related pathways enabling the delivery of enzymes to degrade plant cell walls [22,23,24,25]. Calcium signaling and ligand–receptor interactions may also play key roles in detecting host signals and coordinating infection strategies [26]. The GO analysis highlighted essential biological processes like cellular processes, metabolic functions, and membrane activities, which are crucial for maintaining PWN’s cellular integrity and allowing it to adapt to the host’s challenging internal environment [27,28,29]. The regulation of these processes ensures that PWNs can effectively process nutrients, overcome host defenses, and manage cellular stress. KOG analysis revealed enrichment in signal transduction mechanisms, amino acid and carbohydrate metabolism, and transport. These pathways suggest that PWNs rely on efficient nutrient acquisition and energy metabolism to fuel their growth and reproduction [30,31]. Additionally, signal transduction pathways likely enable PWNs to quickly adapt to environmental stressors and host defenses, ensuring their survival across different regions [27].

Given PWN’s initial invasion in warmer regions of China, we compared genetic variants between populations from cold-temperate (Liaoning) and subtropical (Anhui and Jiangxi) regions. Several high-impact variants were identified in the cold-temperate populations, with candidate genes such as *Bm1-29045* (involved in genetic information processing) [32], *Vha-12*, *Ugt-58*, and *SETMAR* (involved in metabolism and environmental response) [33]. These genes may play crucial roles in PWN’s ability to survive in colder climates by regulating metabolism, ion transport, and cellular homeostasis under cold stress [34,35]. These genetic adaptations suggest that the PWN genome is fine-tuned to endure diverse environmental conditions.

This study provides valuable insights into the genetic background of PWN populations, particularly their ability to adapt to different climates. Understanding these genetic variants is essential for predicting the spread of PWN into regions like northern China, where it was previously thought to be incapable of surviving. Future research should prioritize the use of the genetic markers identified in this study for monitoring and early detection of cold-adapted PWN populations, aiding in the detection of potential new invasions in colder regions. Additionally, gaining a deeper understanding of the genetic basis of PWN adaptation will help inform strategies to prepare forests in at-risk regions, such as developing resistant tree varieties or implementing targeted management practices. Further investigation into candidate genes like *Vha-12* and *Bm1-29045* is necessary to clarify their specific roles in cold adaptation and pathogenicity, enhancing our understanding of how PWN survives and spreads in diverse environments.

## 4. Materials and Methods

### 4.1. Sampling

Samples of indigenous PWN were collected from various regions across China to capture a representative range of geographic and climatic conditions. In the northern region, 11 samples were obtained from different cities within Liaoning Province, which experiences a cold-temperate climate. This area was specifically selected to encompass diverse environmental conditions, including diverse cities and districts. For example, Shenyang, which is situated in the temperate climate zone, experiences significant monsoonal influence year-round, with a mean annual temperature of 8.1 °C and recorded extremes ranging from −28.5 °C to 24.7 °C, making it considerably cooler than most southern Chinese cities. In contrast, 2 samples were collected from the subtropical provinces of Anhui and Jiangxi in southern China. These provinces are characterized by humid subtropical climates with no distinct dry season, representing a warmer climate where PWN invasions have been more frequent.

The selection of these 13 samples was guided by the need to ensure geographic diversity and to account for varying environmental factors across the regions. Sites were chosen to represent a range of climatic conditions, from cold-temperate environments in the north to humid subtropical climates in the south, as well as the presence of host trees susceptible to PWN infection. This approach enabled a comparative analysis of nematode populations in different climatic regions, offering valuable insights into the genetic adaptations of PWN to diverse environmental conditions.

### 4.2. Genomic DNA Isolation, Sequence Library Preparation, and Quality Control

The collected PWNs were processed for DNA extraction at Shenyang Institute of Technology and Southwest Forestry University. The concentration of DNA was measured using the dsDNA BR assay with a Qubit^®^ 2.0 fluorometer (Life Technologies, Carlsbad, CA, USA). Next-generation sequencing libraries were prepared using NovaSeq 6000 Reagent Kit (Illumina Inc., San Diego, CA, USA). Illumina Novaseq6000 (Illumina Inc., San Diego, CA, USA) was used to sequence the single-indexed genomic libraries. Sequencing was performed in paired-end mode with a read length of 150 bp.

### 4.3. Short-Read Preprocessing, Variant Calling, and Filtering

Raw sequencing data were preprocessed using FastQC v0.11.8 [36], to assess sequence quality, including base quality scores, GC content, and adapter contamination. Adapter sequences and low-quality reads (those with a quality score lower than Q20 and reads shorter than 35 nucleotides) were removed using Trimmomatic v0.39 [37]. Clean reads were then mapped to the *Bursaphelenchus xylophilus* reference genome (Genome assembly BXYJv5) using Bwa mem2 v2.2.1 [38]. The resulting SAM/BAM files from the mapping process were sorted by coordinates using Samtools v1.14 [39]. The sorted BAM files obtained were subsequently used for variant calling. Population variants were called using the GATK v4.1.1.0 [40] HaplotypeCaller module with default parameters, along with MarkDuplicates [41] for PCR duplicate removal. Variants with a coverage depth of less than 4 were filtered out using Bcftools v1.9 to minimize potential false positives [42]. Variant density was visualized using the R package circlize v0.4.16 [43].

### 4.4. Population Structure Analysis with Principal Component Analysis and Phylogenetic Tree

To assess the genetic population structure of PWN across different regions, a PCA was conducted on whole SNP data using PLINK v2.0 [44], targeting the six chromosomes. The PCA plots were visualized using the R package ggplot2 v3.5.1 [45]. For the estimation of individual phylogenetic relationships, a genetic distance matrix was generated with PLINK v2.0 [44]. The phylogenetic tree was visualized using the R package APE v5.8 [46].

### 4.5. Annotation Analysis and Functional Enrichment Analyses

Variants were annotated using SnpEff v4.3T [47] to classify SNP/Indel variants based on their genetic locations. For functional annotation, predicted gene-coding variants were aligned to multiple public databases, including Ensembl, NR, NT, COG/KOG, and Uniprot, using NCBI BLAST+ v2.9.0 with an E-value cutoff of 1 × 10^−5^ [48]. GO terms and KEGG pathways for predicted function enrichment analysis of genes and variants were assigned using clusterProfiler v4.10.1 [49]. Only pathways with a *p*-value less than 0.05 were considered to be significantly enriched.

### 4.6. Annotation Analysis of Specific Genetic Variants in Cold-Temperate Regions

Genetic variants between populations from cold-temperate regions (Liaoning province) and subtropical regions (Anhui and Jiangxi provinces) were compared to identify those associated with adaptation to colder climates. To minimize false positives, only genetic variants (SNPs/Indels) present in at least three samples (MAF > 0.1) in colder climates and absent in the subtropical populations were included. Annotation and GO analysis were performed by SnpEff v4.3T [47] and clusterProfiler v4.10.1 [49], with detailed results provided in Supplementary File S7.

High-impact variants were identified based on their genomic locations and variant types, including frameshift variants, stop-gain variants, splice site variants, start-loss variants, and stop-loss variants. These high-impact variants are expected to significantly affect gene function. The retained variants were selected for further analysis to investigate their potential roles.

## 5. Conclusions

This study sampled and analyzed WGS data from 13 indigenous PWN samples collected from both cold-temperate and subtropical regions. Based on identified genetic variants and functional enrichment analyses, we expanded the genetic catalog of PWN populations across distinct ecosystems, thereby providing a genetic basis for their preadaptation to different temperate regions. Notably, several high-impact genetic variants and putative candidate genes were identified in cold-temperate PWN populations, such as *Bm1-29045*, *Vha-12*, *Ugt-58*, and *SETMAR*. These genes are involved in critical functions like genetic information processing, metabolism, and environmental response, which may facilitate PWNs’ adaptation to colder climates. These genetic findings enhance our understanding of PWNs’ spread in China and offer critical insights for future management and mitigation strategies aimed at controlling this invasive species.

## Figures and Tables

**Table 2:** Relevant genes associated with high-impact variants (SNPs and Indels) in cold-temperate (Liaoning province) compared to subtropical (Anhui and Jiangxi provinces) populations.

Gene Symbol	Description	Annotation	Variant Type
*Bm1-29045*	TKR78190.1 hypothetical protein	Genetic information processing; Translation	Stop gained, frame shifted
*F59C6.8*	TKR58295.1 hypothetical protein	− *	Frameshift variant
*Tag-151*	KAE9554954.1 hypothetical protein	−	Frameshift variant
*Vha-12*	KHJ82218.1 hypothetical protein	Metabolism; Environmental information Processing; cellular processes	Frameshift variant
*At1g67520*	VDN83815.1 unnamed protein product	−	Frameshift variant
*Ugt-58*	KAE9553687.1 hypothetical protein	Metabolism	Frameshift variant
*OatA*	XP_003093275.1 hypothetical protein	−	Frameshift variant
*Lys-3*	XP_024506972.1 Glycoside hydrolase	−	Frameshift variant
*SETMAR*	AAZ67092.1 transposase	Metabolism	Frameshift variant

*: Dash indicates no relevant annotation available.

## Data Availability

The raw full-length sequencing data (in FASTQ format) have been submitted to the European Nucleotide Archive (ENA) under the project accession number PRJEB78855 (ERP163091).

## Supplementary Materials

The following supporting information can be downloaded at https://www.mdpi.com/article/10.3390/ijms251910492/s1.
